# The Micro-Organism of Whooping-Cough

**Published:** 1883-04

**Authors:** 


The Micro-Organism of Whooping-Cough.—Dr. Carl Burger (*Berliner Klin. Woch.*) describes an organism which he has found in the sputa of patients with whooping-cough, which are so constant in their appearance in this disease, while never to be seen under other circumstances, that he believes there must be some etiological reason for their presence, particularly since the intensity of the disease depends upon the number of the organisms present. He has not yet reported the results of the culture-experiments with which he is at present occupied.

These organisms are readily detected by simple staining with watery aniline solutions, and may be prepared in the same manner as employed by Koch in the case of the bacillus tuberculosis. Dr. Burger describes them as ovoidal rods, about twice as long as broad, with a slight constriction around their middle; occasionally they are met in chains and groups, and by their difference in size and shape can be readily distinguished from the spores of *Leptothrix buccalis.*—*Medical News.*

